# C-tactile afferent stimulating touch carries a positive affective value

**DOI:** 10.1371/journal.pone.0173457

**Published:** 2017-03-10

**Authors:** Ralph Pawling, Peter R. Cannon, Francis P. McGlone, Susannah C. Walker

**Affiliations:** 1 Research Centre for Brain & Behaviour, School of Natural Sciences & Psychology, Liverpool John Moores University, Liverpool, United Kingdom; 2 School of Psychology, Massey University, Auckland, New Zealand; 3 Institute of Psychology, Health & Society, University of Liverpool, Liverpool, United Kingdom; University of Ottawa, CANADA

## Abstract

The rewarding sensation of touch in affiliative interactions is hypothesized to be underpinned by a specialized system of nerve fibers called C-Tactile afferents (CTs), which respond optimally to slowly moving, gentle touch, typical of a caress. However, empirical evidence to support the theory that CTs encode socially relevant, rewarding tactile information in humans is currently limited. While in healthy participants, touch applied at CT optimal velocities (1-10cm/sec) is reliably rated as subjectively pleasant, neuronopathy patients lacking large myelinated afferents, but with intact C-fibres, report that the conscious sensation elicited by stimulation of CTs is rather vague. Given this weak perceptual impact the value of self-report measures for assessing the specific affective value of CT activating touch appears limited. Therefore, we combined subjective ratings of touch pleasantness with implicit measures of affective state (facial electromyography) and autonomic arousal (heart rate) to determine whether CT activation carries a positive affective value. We recorded the activity of two key emotion-relevant facial muscle sites (zygomaticus major—smile muscle, positive affect & corrugator supercilii—frown muscle, negative affect) while participants evaluated the pleasantness of experimenter administered stroking touch, delivered using a soft brush, at two velocities (CT optimal 3cm/sec & CT non-optimal 30cm/sec), on two skin sites (CT innervated forearm & non-CT innervated palm). On both sites, 3cm/sec stroking touch was rated as more pleasant and produced greater heart rate deceleration than 30cm/sec stimulation. However, neither self-report ratings nor heart rate responses discriminated stimulation on the CT innervated arm from stroking of the non-CT innervated palm. In contrast, significantly greater activation of the zygomaticus major (smiling muscle) was seen specifically to CT optimal, 3cm/sec, stroking on the forearm in comparison to all other stimuli. These results offer the first empirical evidence in humans that tactile stimulation that optimally activates CTs carries a positive affective valence that can be measured implicitly.

## Introduction

In highly social species, across the lifespan, touch plays a central role in the formation and maintenance of relationships. Between an infant and caregiver, within primate hierarchies and in romantic relationships, tactile interactions are rewarding, buffer physiological and psychological responses to stress and ultimately enhance well-being [1–5]. For example, parental touch is a key regulator of an infant’s physiological and behavioural arousal [2] and in adults, supportive physical contact from a spouse or partner has been shown to modulate physiological responses to an acute stressor to a significantly greater degree than verbal support [5]. Indeed, despite the dominance of vision and audition in human social communication, studies have shown that we can also reliably communicate emotions through tactile interactions [6]. In fact, it has been proposed that humans may rely more heavily on such non-verbal forms of communication in times of stress [7]. Despite this centrality of touch to our social world, to date, little attention has been paid to the neurophysiological basis of its beneficial effects.

C-Tactile afferents (CTs) are unmyelinated low threshold mechanoreceptors innervating the skin of mammals [8,9]. In humans, their response characteristics have been elegantly mapped using single-unit microneurography recordings, establishing that CTs respond optimally to a low force and velocity skin temperature stimulus moving across their receptive field [10,11]. During microneurography sessions, CTs are encountered as often as myelinated Aβ afferents in the hairy skin (face & arm). However, they have never been recorded from nerves innervating the glabrous skin of the palms [11,12,13, though see 14]. In contrast to Aβ afferents, which display a linear relationship between firing frequency and stimulus velocity, the response curves of CTs are best described by an inverted U-shaped function, responding most strongly to a stimulus moving at between 1-10cm/sec [12]. Intriguingly, there is a positive correlation between CT firing frequency and people’s ratings of touch pleasantness [11,15]. That is, the stimulus parameters which excite CTs most strongly are also those which participants subjectively rate as most pleasant. In support of their affective as opposed to sensory function, neuroimaging studies in Aβ deafferented patients and healthy participants have shown that, consistent with other C-fibres signalling pain and itch, CTs project to the posterior insula cortex, a region hypothesised to play a role in maintaining the homeostatic condition of the body [16,17].

Given the apparent biological relevance of the tactile stimuli CTs encode, it has been posited that they have evolved to signal the rewarding value of social tactile interactions. In support of this Social Touch Hypothesis [8,18], a recent observational study reported that, when asked to caress their infant or partner, participants spontaneously stroked at velocities that, on average, fell within the CT optimal range [19]. Furthermore, consistent with a soothing or calming function, Fairhurst et al [20] reported that stroking touch delivered at CT optimal velocity to the forearm of 9-month old infants produced a selective decrease in heart rate, that was not seen in response to either faster or slower, CT non-optimal, stroking. Rodent studies provide direct evidence that CT activating touch is rewarding, as selective activation of C-low threshold mechanoreceptors (C-LTMs -the rodent homologue of CTs) using pharmacogenetics lead to the formation of conditioned place preference [21].

However, an apparent challenge to the Social Touch Hypothesis comes from the observation that gentle stroking touch applied to the glabrous skin of the palm, where CTs are not thought to innervate, is reliably rated as pleasant, indicating that CTs alone are not responsible for signalling the affective value of touch [22,23]. It has been suggested that while activation of CTs carries an innate rewarding value, the perceived pleasantness of touch on glabrous skin is the result of secondary reinforcement—i.e. the touch has become associated with the pleasant context it is experienced within [9].

A further explanation may be that subjective ratings are not sufficient to discriminate the differences between CT and non-CT activating touch. For example, McGlone et al [24] reported that while there was no difference in perceived intensity and pleasantness of touch applied either to the CT innervated volar forearm or the glabrous skin of the palm, the language participants used to describe their perception of touch differed across sites. Specifically, emotional descriptors, such as “comfort”, received significantly higher ratings in response to touch on the forearm than touch on glabrous skin, which generated more sensory (descriptive) ratings (e.g. fluffy). This psychophysical finding is consistent with the accompanying imaging data that revealed significantly greater activation in both posterior insular and orbitofrontal cortices to stroking on the forearm when contrast with the same brush stroking on the palm. Additionally, psychophysical tests in neuronopathy patients lacking large myelinated afferents, but with intact C fibres, indicate that the conscious sensation elicited by stimulation of CTs is rather weak [17]. Thus, in order to directly test the Social Touch Hypothesis, measures of affective experience are required that go beyond explicit subjective evaluations.

Facial electromyography (EMG) provides an implicit measure of affective response that is relatively independent of autonomic arousal [25]. Indeed, EMG has been used successfully across a range of sensory modalities as an index of both positive and negative responses to stimuli [26–29]. Unpleasant stimuli or experience is associated with an increase in activity of the corrugator supercilii (frown) muscle, while processing of pleasant stimuli is associated with greater activity of the zygomaticus major (smile) muscle [26]. It has also been demonstrated that EMG can be used to discriminate affective responses to perceptually similar sensory stimuli that subjective ratings of pleasantness do not. For example, an olfactory study recently reported greater zygomaticus activity in participants exposed to the odour of sweat samples collected from donors in a positive emotional state than perceptually indistinguishable samples collected while donors were in a negative or neutral state [28]. This suggests that EMG is an effective tool for differentiating experiences that influence behaviour, but are below an individual’s threshold of conscious awareness.

In the present study, we used convergent physiological measures of affect (facial EMG) and arousal (heart rate), along with subjective self-report ratings of pleasantness, to compare participants’ experiences of touch stimulation of the arm and palm. We tested two hypotheses: first, that CT optimal stimulation of the forearm would result in increased positive affect-related facial muscle activity and reduced heart rate, compared with higher velocity (CT non-optimal) stroking; and second, that there would be physiological but not subjective discrimination of CT-optimal stroking between the arm and the palm.

## Methods

### 2.1 Participants

Twenty-nine participants (18 female; 26 self-reported being right handed) were recruited via poster adverts from the student and staff population at Massey University, NZ, and from the general public in the Albany area of Auckland, NZ. The mean age of the sample was 30.4 years (SD = 16.4 years). The study was performed according to the Declaration of Helsinki on Biomedical Research Involving Human Subjects and was granted ethical approval by Massey University ethics committee. All participants gave written informed consent and were compensated for their time with shopping vouchers.

### 2.2 Measures

All psychophysiological measures were collected using a Biopac MP150 system, sampled at 2000Hz, and recorded using AcqKnowledge (Version 4.2, Biopac Systems Inc., CA, USA). The data were time-locked to the onset of touch via parallel port signals sent from the experimenter’s computer to the physiological recording equipment. Offline filtering and heart beat detection were also conducted in AcqKnowledge before the data were exported into SPSS (Version 23, IBM Corp, USA, NY).

#### 2.2.1 EMG

EMG data were collected from the zygomaticus major and corrugator supercilii muscles. Bipolar placements of shielded, 4mm Ag/AgCl electrodes were attached over the muscle sites with adhesive discs, and an unshielded ground electrode placed just below the hairline in the center of the participant’s forehead. The electrode placement and site preparation procedures followed the guidelines set out in [30]. The EMG data were filtered online between 1Hz and 5000Hz, and a 50Hz notch filter was applied. The data were amplified with a gain of 5000X. Offline the data were passed through a 20-400Hz bandpass filter [31].

#### 2.2.2 Heart rate

Self-adhesive, pre-gelled disposable ECG electrodes were placed on the skin just above the participant’s left hip and just below their right clavicle, with a ground electrode placed below the left clavicle. The sites were wiped with an alcohol swab prior to attachment. ECG data were filtered online between 0.1Hz and 150Hz, and with a notch filter applied at 50Hz. Data were amplified at a gain of 500X. Offline the data were bandpass filtered between 0.1Hz and 30Hz.

#### 2.2.3 Delivery of tactile stimuli and pleasantness ratings

**Fig 1:**
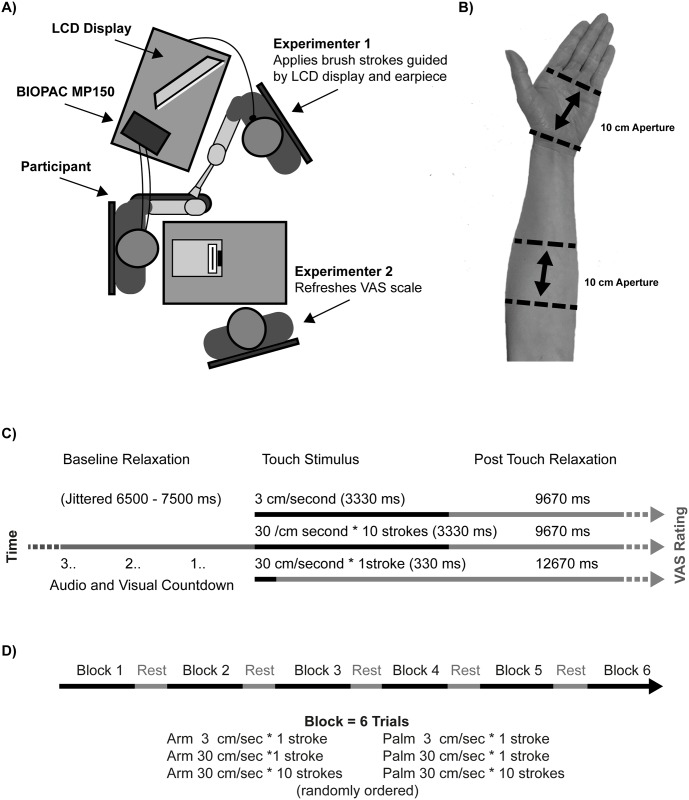
**A*)*** The layout of the experiment showing the seating positions of the participant and the two experimenters. **B)** The apertures on the palm and volar forearm at which touch was applied. **C)** The time course of each trial type, showing the common duration of jittered relaxation period prior to touch, the duration of the three conditions of touch stimulus that were applied to the palm and volar forearm, and the duration of the post-touch period, prior to the participant making their VAS rating. **D)** The experimental design, illustrating the six blocks and the six trial types presented in a random order in each block.

The layout of the laboratory in which the experiment was conducted can be seen in Fig 1A (the layout was adjusted for left handed participants to allow them to make their VAS ratings with their dominant hand). Experimenter 1 sat in front of a computer display which they and Experimenter 2 could see, but the participant could not. This computer controlled the timing and randomization of the task via a script run using EPrime 2.0 Professional software [32].

Experimenter 1 delivered touch stimuli to the participant’s palm and volar forearm, using a soft goat hair brush, with a width of 44mm. An aperture of 10cm, within which the stroking was delivered, was marked at each location using surgical tape strips (see Fig 1B). Touch was delivered at speeds of 3cm/sec * 1 stroke (CT optimal), 30cm/sec * 1 stroke (CT non-optimal matched for stroke number) and 30cm/sec * 10 strokes (CT non-optimal matched for duration). Experimenter 1 was aided in accurately timing the touch by a visual metronome, presented on their display, which also provided a prompt as to which speed / number of repetitions was to be used in the current trial. Single strokes (3cm/sec * 1 and 30cm/sec * 1) were delivered in a proximal to distal direction, with the 10 strokes at 30cm/sec delivered from proximal to distal and back again five times (for time courses see Fig 1C). Experimenter 1 practiced using the randomized order conditions and metronome until they could reliably deliver a stroking stimulus of consistent force (approx. 0.3N) and velocity.

The task consisted of 36 experimental trials and started with 3 practice trials. The 36 trials were divided into 6 blocks of 6 trials, with each block consisting of one stimulation of each speed condition (3cm/sec, 30cm/sec * 1, 30cm/sec * 10), applied to each location (palm, volar forearm), in a random order (see Fig 1D). The practice trials were 3 randomly selected combinations of location and speed.

Immediately after each trial the participant rated the touch they had just received using a 200mm visual analogue scale (VAS) with anchor points ‘Unpleasant’ and ‘Pleasant’. VAS scales ranging from unpleasant to pleasant have been shown in previous studies to provide a reliable and sensitive measure of differences in affective touch perception across stimuli and locations [15,33,34].

### 2.3 Procedure

The experimenters (both males in their thirties) greeted the participant, provided them with a verbal and written briefing and collected written consent. The participant sat in a comfortable chair with arm rests, while the experimenters attached the electrodes, after which the participant relaxed for two minutes prior to the practice trials. Throughout the touch task the participant sat with their non-dominant arm placed palm up on the arm rest of the chair. They could move the arm between blocks and used their dominant hand to complete the rating scales, placed on a table in front of them. Experimenter 1 sat in front of the participant, and Experimenter 2 to one side (see Fig 1A).

Before initiating each trial with a key press, Experimenter 1 instructed the participant to close their eyes and flatten the palm of their non-dominant hand. The intention of having the participant close their eyes was to remove any visual distractions during the touch stimulus. Experimenter 1’s computer then prompted the type and location of touch for the trial and displayed a visualization of the touch aperture, onto which the metronome would be imposed. The trial began with a jittered baseline period ranging between 6500ms and 7500ms, ending in a visual “3, 2, 1” countdown presented on screen and accompanied by three beeps played to Experimenter 1 through an ear piece. The metronome was then activated and Experimenter 1 delivered the relevant touch stimulus. The touch at 3cm/sec and 30cm/sec * 10 lasted approximately 3330ms, the touch at 30cm/sec * 1, approximately 330ms. This time period was subtracted from a 13000ms post stroke rest period, during which the participant remained eyes closed and psychophysiological responses continued to be recorded. This resulted in a trial window of 13000ms from stroke onset to the instruction from the experimenter for the participant to open their eyes (see Fig 1C). Immediately after opening their eyes the participant made their pleasantness rating, and Experimenter 2 then refreshed the paper scale. The next trial was initiated when the participant stated they were relaxed and between blocks a 30sec rest period was enforced during which the participant could relax / move / stretch, with their eyes open.

### 2.4 Data Analysis

#### 2.4.1 VAS ratings data

The VAS ratings were measured by hand, with a value of 1 applied for a mark made at the far left end of the scale, ranging to a value of 200 for marks made at the far right end. The VAS ratings were analysed using a repeated measures ANOVA, with within subject factors of Location (Arm & Palm) and Stroke Type (3cm/sec, 30cm/sec * 1 & 30cm/sec * 10).

#### 2.4.2 Heart rate data

Each participant’s electrocardiogram (ECG) trace was passed through a peak-finding algorithm in AcqKnowledge (Version 4.2, Biopac Systems Inc., CA, USA) to identify R-peaks. An automated event-finding algorithm then converted these into a train of inter-beat intervals (IBIs) expressed as the number of seconds between each R-peak and the previous R-peak. These data were then entered into SPSS (Version 23, IBM Corp, USA, NY) and custom scripts were used to divide the data into trial epochs. On a trial by trial basis, the mean of the two IBIs immediately prior to touch onset was calculated as a baseline. Baselines containing IBIs of a value falling more than three standard deviations from the mean of all a participant’s baseline IBIs were identified and replaced by the mean of all IBIs from that participant’s non-artefactual baselines. The first 5 beats post-touch onset were then expressed as a change in seconds from the baseline for the trial, meaning that a negative value represents heart rate acceleration and a positive value heart rate deceleration. Any trials containing IBI change scores that exceeded four standard deviations from the mean of all change scores were removed from the analysis, an average of 3% of trials per participant. Analysis was conducted on the mean change in IBI across the five beats for each trial. Change scores were entered into a repeated measures ANOVA with the within subjects factors of Location (Palm & Arm) and Stroke type (3cm/sec, 30cm/sec * 1 & 30cm/sec * 10).

#### 2.4.3 EMG data

The EMG data for both muscle sites were processed with custom scripts. All data processing was completed on a participant-by-participant basis, and separately for the two muscle sites, by an experimenter blind to trial type. The data were full-wave rectified, divided into trials and down-sampled by calculating the mean amplitude of the signal for every 100ms period after trial onset. A pre-trial baseline level of muscle activity was calculated for every trial, so that muscle activity elicited by the touch stimuli could be expressed as a change from baseline. Baselines were achieved by calculating, on a trial by trial basis, the mean level of muscle activity between 5500ms and 6500ms after the trial onset (when the participant sat, eyes closed, awaiting the touch stimulus), referred to now as the ‘rest period’. This period was chosen because across participants it demonstrated a reliably low level of variation in the activity of both muscles, and best represented resting state. For each trial, every 100ms epoch after the onset of touch was then expressed as a percentage change score from that trial’s baseline, so that positive values represent an increase in muscle activity of n-percent, and negative values a decrease of n-percent.

To account for non-experimental movements that might cause extreme values, before the baselines were calculated, a grand mean was generated for all data (100ms epochs) contained within the rest period across all trials. Any rest period that contained an epoch falling more than three standard deviations from this mean was marked as an artefact. For rest periods identified as containing artefacts, instead of generating a baseline via the mean of the period’s own activity, the baseline was represented by the grand mean of activity across all rest periods that did not contain artefacts. Next, trials containing very extreme percent change scores (> 500%) were removed from analysis, as these levels of change occurred only when a participant performed actions such as yawning and coughing. This resulted in the removal of an average of 7% of trials for zygomaticus data, and 2% for corrugator data across participants. The data were then divided into two time-bins for analysis by calculating the mean activity during the first 3500ms after touch onset, which was considered the Touch Period, and between 3500ms and 13000ms which was considered the Post-touch Period. The data from the zygomaticus and corrugator muscles were analysed using separate repeated measures ANOVAs with the factors of Location (Palm & Arm), Stroke Type (3cm/sec, 30cm/sec * 1 & 30cm/sec * 10) and Time (Touch & Post-touch).

## Results

### 3.1 VAS ratings

**Fig 2:**
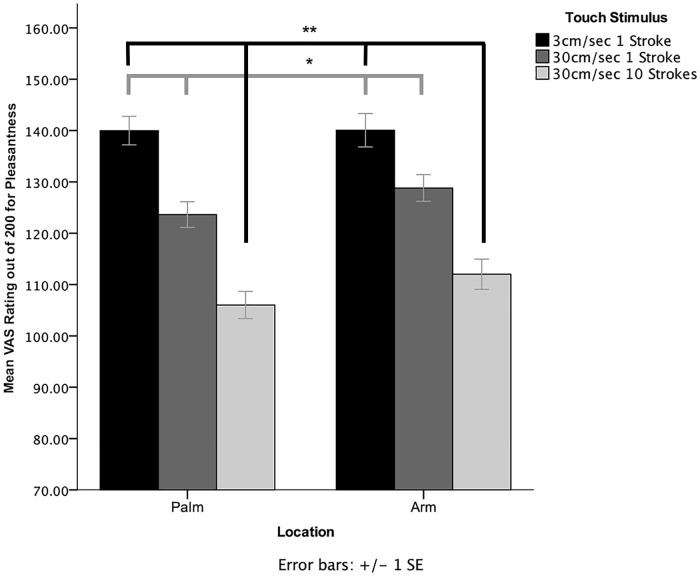
Mean pleasantness ratings (+/- 1 SE) of touch applied to the palm and volar forearm, for stimulations at CT-optimal speed (3cm/sec * 1 stroke), and CT non-optimal speeds (30cm/sec * 1 stroke, and 30cm/sec * 10 strokes). Black lines indicate the significant difference in pleasantness ratings for touch at 3cm/sec compared to 10 strokes of touch at 30 cm/sec, and grey lines the significant difference for touch at 3 cm/sec versus 1 stroke at 30 cm/sec. Neither effect interacted with the location of touch. *p<0.05, **p<0.01.

The analysis showed a significant main effect of Stroke Type, *F*(2,56) = 17.2, *p* < .001, partial η^2^ = .38. Post hoc contrasts revealed that touch at 3cm/sec was rated as significantly more pleasant than both one (*p* = .01) and ten (*p* < .001) strokes of 30cm/sec touch. There was no significant main effect of Location, *F*(1,28) = 1.04, *p* = .32, partial η^2^ = .036, nor an interaction between Location and Stroke Type, *F*(2,56) = 1.5, *p* = .23, partial η^2^ = .05. Thus, stroking delivered at 3cm/sec was rated more positively than stroking delivered at 30cm/sec irrespective of whether it was applied to the CT innervated forearm or the glabrous skin of the palm (see Fig 2).

### 3.2 Heart rate

**Fig 3:**
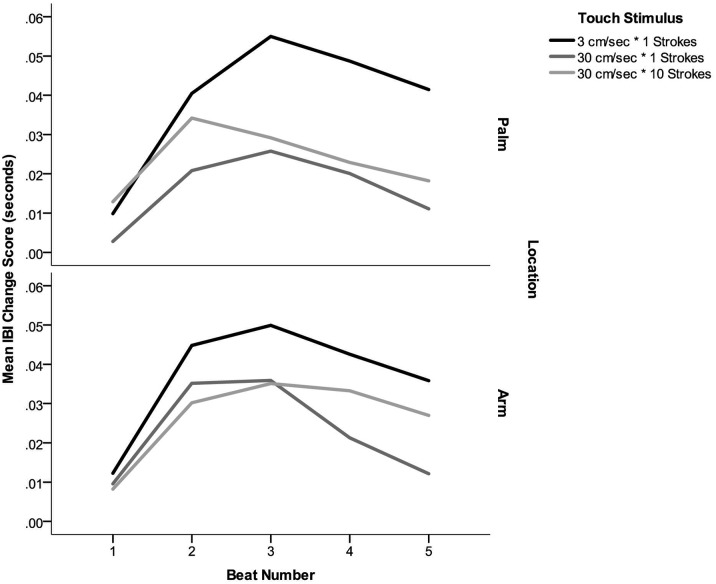
Heart rate responses, represented as change in IBI to CT-optimal (3cm/sec * 1 stroke) and CT non-optimal (30cm/sec * 1 & 10) touch stimuli, applied to the palm (top panel) and arm (bottom panel). Change scores represent change from baseline in seconds, with positive values representing longer IBIs, and thus a slowing of heart rate from baseline levels.

There was a significant effect of Stroke Type, *F*(2,50) = 9.1, *p* < .001, partial η^2^ = 2.7. Contrasts revealed that stroking at 3cm/sec elicited significantly greater heart rate deceleration than either a single stroke at 30cm/sec (*p* < .001) or ten strokes at 30cm/sec (*p* = .008). There was no significant effect of Location, *F*(2,25) = .48, *p* = .50, partial η^2^ = .019, or interaction between Location and Stroke Type, *F*(2,25) = 1.2, *p* = .32, partial η^2^ = .045. Thus, irrespective of whether it was applied to CT innervated forearm skin or the glabrous skin of the palm, stroking at 3cm/sec produced a significantly greater decrease in heart rate than faster bouts of touch (see Fig 3).

### 3.3 EMG

The first EMG analyses compared the effects of Stroke Type (3cm/sec, 30cm/sec * 1 & 30cm/sec * 10), Location (Palm & Arm) of touch, and Time (Touch & Post-touch) in terms of their influence on positive and negative affective muscle responses. In the analysis of the corrugator there was no significant main effect of Stroke Type, *F*(1,28) = .62, *p* = .54, partial η^2^ = .022, or interaction between Stroke Type and Location, *F*(1,28) = 1.092, *p* = .34, partial η^2^ = .038, or Stroke Type and Time, *F*(1,28) = .20, *p* = .82, partial η^2^ = .007. Similarly in the zygomaticus there was no main effect of Stroke Type, *F*(1,28) = .75, *p* = .48, partial η^2^ = .026, or interaction between Stroke Type and Location, *F*(1,28) = 2.1, *p* = .13, partial η^2^ = .070, or Stroke Type and Time, *F*(1,28) = 1.3, *p* = .28, partial η^2^ = .044. All other main effects and interactions, not involving Stroke Type were non-significant, with all values of *F* < 2.2.

**Fig 4:**
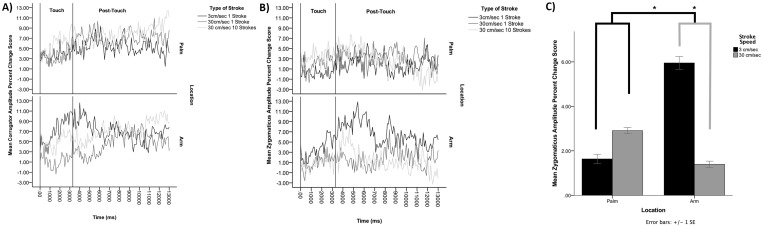
***A)*** The time course, in milliseconds, of activity in the corrugator muscle in response to touch applied to the palm (top panel) and arm (bottom panel) at CT-optimal speed (3cm/sec * 1 stroke) and CT non-optimal speeds (30cm/sec * 1 & 10 strokes). Activity levels are percent amplitude changes from baseline, and the plot is divided into ‘Touch’ representing the period during which touch was applied, and ‘Post-Touch’ representing the period afterward where the participant contemplated the touch prior to making their rating. **B)** The same time course displayed for the zygomaticus muscle. **C)** Bar-chart displaying the interaction effect (*p<0.05) in the zygomaticus muscle between Location and Speed of touch (black lines), and the significant effect of Speed of touch on the arm (grey lines).

In order to increase the power of our analysis, a direct comparison of CT touch versus non-CT touch was made by collapsing across the two different versions of touch at 30cm/sec, and comparing this combined non-CT stimulus with touch delivered at 3cm/sec. Again, separate repeated measures ANOVAs were conducted for the two muscle sites, this time with the factors of Location (Palm &Arm), Speed (3cm/sec & 30cm/sec), and Time (Touch & Post-touch). In the corrugator muscle there were again no significant main effects or interactions (see Fig 4A), with all values of *F* < 1.8. However, the analysis of the zygomaticus muscle revealed a significant two way interaction between Location and Speed, *F*(1,28) = 4.3, *p* = .048, partial η^2^ = .132. All other main effects and interactions were non-significant, with *F* values < 1.5. Paired samples t-tests revealed that whilst there was no significant difference in zygomaticus activity in response to the two speeds of stroking to the palm, *t*(28) = -.53, *p* = 0.60, activity was significantly greater in response to stroking at 3cm/sec than to stroking at 30cm/sec to the forearm, *t*(28) = 2.4, *p* = 0.02. Thus, consistent with our hypothesis, touch which selectively targets CTs, (i.e. applied at 3cm/sec to CT innervated forearm skin) induced greater activity in the zygomaticus muscle, indicative of a positive affective response, than touch applied at faster velocities or to the palm of the hand (see Fig 4B and 4C).

In order to ensure that the significant Speed x Location interaction identified in the zygomaticus muscle does reflect a CT specific effect, rather than being driven by differences in duration of the tactile stimuli, we conducted a further analysis by running an ANOVA on each muscle site, which compared only the 3 cm/second and 10*30 cm/second stimuli, using the same accompanying factors of Location (Arm v Palm), and Time (Touch v Post Touch). Again, this revealed no significant main effects or interactions in the corrugator. However, in the zygomatics the interaction between Location and Speed was significant, *F*(1,28) = 4.2, *p* = .049, η2 = .131. Whilst post-hoc t-tests comparing the two speeds of touch at the palm, *t*(28) = -.48, *p* = .63, and the arm, *t*(28) = 1.4, *p* = .16, both failed to reach significance, the stability of the interaction effect, and trend at the arm, support the notion that the zygomaticus effect is velocity specific and does not merely reflect differences in the duration of the stimuli applied. To be thorough, we conducted the same analysis in the zygomaticus, between touch at 3 cm/second and 1*30 cm/second, which revealed no significant interaction between Location and Speed, *F*(1,28) = 2.1, *p* = .16, η2 = .070. Taken together, these findings suggest that the difference in implicit affective response between the CT and non-CT stimuli was driven by the differing velocities of the two stimuli that were matched for duration.

## Discussion

Our data show that dynamic stroking touch applied to the forearm, but not the palm, at CT optimal velocity elicited significantly greater activation in the zygomaticus major muscle of the cheek than faster strokes that did not optimally activate CTs. Given activation of this muscle has previously been associated with positive affective reactions [26,28,29], this finding supports our hypothesis, demonstrating for the first time that consistent with a role in signaling the rewarding value of social tactile interactions, activation of CTs generates an implicit emotional response.

Physiologically, and in line with findings from a previous study [20], we found that CT activating touch elicited a significantly larger heart rate deceleration response than faster stroking. Heart rate deceleration has been associated with appetitive motivation [25], suggesting that this touch represented a more powerfully rewarding stimulus. However, in this study we extended previous work by also examining the effects of different speeds of stroking on the glabrous skin of the palm, where CTs are not thought to innervate. Here too we saw a reduction in heart rate to slow versus fast stroking touch that did not differ statistically from that seen for touch to the arm. This partial dissociation in response to speed but not location of stimulation was matched by the self-report ratings of touch pleasantness, where slow stroking delivered to both the hairy skin of the arm and the glabrous skin of the palm was rated as more pleasant than the faster CT non-optimal stimulus.

The lack of difference in pleasantness ratings between arm and palm is consistent with previous studies [24,35,though see 11,15]. Not only is touch to the palm perceived as pleasant [22] fMRI studies have also shown it elicits responses within the orbitofrontal cortex (OFC) [36,37]. Though to date the role of Aβ afferents in signaling the pleasant sensations of touch have not been widely explored, contrast analysis of CT targeted touch on hairy versus glabrous skin shows higher activations in posterior-insular and OFC to the CT targeted touch and in somatosensory cortices for glabrous touch [24]. This processing in limbic regions is supportive of the hypothesis that CT touch has an innate rewarding value while pleasant sensations elicited by touch on glabrous skin are learned. Indeed, Löken et al [35] reported that within a testing session ratings of touch pleasantness on the CT innervated forearm reliably influenced pleasantness ratings on subsequent trials where touch was applied to the palm of the hand, an effect they interpreted as reflecting positive priming. Given the randomized block design used in the present study it is entirely possible this order effect also underlies our subjective ratings. Interestingly, Löken et al [35] note that the experience of stroking on the palm does not influence ratings on the forearm, consistent with the hypothesis that there is a weaker affective impact of this stimulus. This finding is supported by studies using a touch perception questionnaire that reliably shows touch on hairy skin has a greater affective value than touch on the glabrous skin of the palm, which is assigned more sensory than emotional verbal descriptors [24,38].

Previous studies have also reported that affiliative touch, delivered in a range of contexts, leads to a general increase in parasympathetic nervous system activity as indexed by a reduction in heart rate and blood pressure [5,39,40]. Neurochemically, this reduction in arousal in response to cutaneous stimulation is hypothesized to be mediated by the release of oxytocin [41]. While the nature of the cutaneous nerves underpinning these observed effects has received little attention, CTs, given their response characteristics, present as a strong candidate. However, our finding that heart rate was also reduced in response to stimulation of the palm challenges this hypothesis. Both cardiac deceleration and moderate increases in skin conductance (reflecting increased perspiration) are classic physiological measures of orienting, indicating sensory appraisal which involves co-activation of both sympathetic and parasympathetic systems [25]. Thus, it seems likely that the deceleration in heart rate seen in the present study in response to the brief sensory stimulus, on both the palm and arm, is a reflexive orienting response to these two perceptually similar stimuli that are neither highly arousing nor highly aversive [25]. Consistent with this interpretation, a previous study reported that CT activation through gentle stroking touch on the hairy skin of the forearm elicited a sympathetic response, measured by skin conductance level, despite its weak perceptual impact [42]. In contrast to autonomic measures which vary with the intensity of a sensory stimulus, responses recorded with facial EMG reflect the true affective value of the stimulus [26]. Of the three measures used in the present study it was facial EMG which dissociated the CT optimal stimulus from the others, suggestive of an innately positive affective value.

Further research is needed in order to fully elucidate the autonomic and motivational consequences of CT activating touch, in comparison to non-CT directed cutaneous stimulation. As with other C-fiber based interoceptive signals that provide information about the state of the tissues of the body, CTs are likely to project via the lamina I spinothalamic tract terminating in the posterior insula cortex [43,44, though see 45,46]. Such inputs have been described as generating homeostatic emotions (thirst, hunger, fatigue) which result in a sensation and a motivational drive [44]. The pleasure derived from a sensory stimulus that can return the body to its homeostatic set-point has long been recognized [47]. If, as the Social Touch Hypothesis suggests, CTs have evolved to signal the rewarding value of affiliative tactile interactions, behaviorally their activation is perhaps more likely to drive quiescence (inactivity), than motor readiness [48,49].

Of course the affective value of a sensory stimulus is entirely dependent upon the current internal state of the organism, a phenomena which Cabanac referred to as *alliesthesia* [47]. As an innate neural signal cueing the presence of social support, one would anticipate that CT mediated touch should aid emotion regulation and buffer individuals from stress [50,51]. Future work should test this empirically by determining whether targeted stimulation of CTs can reduce physiological indexes and behavioral consequences of stress.

In conclusion, the findings from the present study provide the first behavioral evidence in humans that CT activating touch carries a positive affective value that can be measured implicitly, thus providing empirical support for the Social Touch Hypothesis [12,18]. Further work is needed to fully elucidate the specific autonomic, neurochemical and behavioural consequences of CT activating touch and how these relate to a person’s affective state.

## Supporting information

S1 File EMG. (SAV)

S2 File Heart rate. (SAV)

S3 File Ratings. (SAV)
